# Advances and challenges in the treatment of myelodysplastic syndromes

**DOI:** 10.1186/s40164-025-00678-9

**Published:** 2025-06-18

**Authors:** Rohit Thalla, Ryan Mack, Jorgena Kosti-Schwartz, Peter Breslin, Jiwang Zhang

**Affiliations:** 1 Oncology Institute, Cardinal Bernardin Cancer Center, Loyola University Chicago Medical Center, Maywood, IL 60153 USA; 2 Department of Cancer Biology, Loyola University Chicago Medical Center, Maywood, IL 60153 USA; 3 Department of Medicine, Loyola University Chicago Medical Center, Maywood, IL 60153 USA; 4 Departments of Biology and Molecular/Cellular Physiology, Loyola University Chicago, Maywood, IL 60153 USA

**Keywords:** MDS, Treatments, Challenges, Innate immunity

## Abstract

Myelodysplastic syndromes (MDS) is a heterogeneous group of pre-leukemic diseases characterized by peripheral blood cytopenia, morphologic dysplasia, and an increased risk of transformation to leukemia. MDS develop from genetically mutant clonal hematopoietic stem and progenitor cells (HSPCs) which have defects in generating mature functional blood cells due to impaired differentiation and/or survival activities. In addition, mutant HSPCs also inhibit the generation of new blood cells from remaining healthy HSPCs. Thus, the complete elimination of mutant HSPCs is the optimal goal for MDS treatment. However, most current therapies for MDS are little more than palliative, primarily addressing cytopenia-related symptoms and improving the quality of life. Only the hypomethylating agents (HMA) lenalidomide and imetelstat reduced the mutational burden, and then only in a small subset of cases. Many HMA-based combination therapies failed to show benefits superior to single-agent HMA treatment in clinical trials. At the present time, allogeneic hematopoietic stem cell transplantation (allo-HSCT) is still the only cure for the minority of qualified patients who have HLA-matched donors. Novel effective treatments are urgently needed. Here we summarize the current standard therapeutic approaches for MDS patients and discuss major advances in MDS research and treatments. We also discuss major challenges and potential solutions to overcome these challenges for future MDS research and drug development.

**Supplementary Information:**

The online version contains supplementary material available at 10.1186/s40164-025-00678-9.

## Highlights

1. Standard therapies for MDS are merely palliative, primarily addressing symptoms.

2. Newer therapies selectively target mutant clones, thus reducing disease bunden.

3. Combining standard and newer treatment strategies is promising but poses toxicity challenges.

4. Targeted and personalized therapies are needed for this heterogeneous group of diseases.

**Supplementary Information:**

The online version contains supplementary material available at 10.1186/s40164-025-00678-9.

## Background

Myelodysplastic syndromes (MDS), also called myelodysplastic neoplasms, is a heterogeneous group of bone marrow (BM) failure diseases originating from clonal hematopoietic stem cells (HSCs) characterized by persistent peripheral blood (PB) cytopenia, morphologic dysplasia, and a high risk of transformation to acute myeloid leukemia (AML) [1, 2]. The incidence rate for MDS is 4 cases per 100,000 people with a median age at diagnosis of ~ 70 years; the incidence increases to 25 per 100 000 persons aged 65 years and older [1, 3]. BM and PB cells from MDS patients contain an average of 3–4 genetic abnormalities [4]. HSCs and progenitor cells (HSPCs) harboring genetic abnormalities comprise 20–80% of BM HSPCs in MDS patients and display defects in the generation of mature blood cells due to their impaired differentiation and survival [5]. The mutant HSPCs induce an inflammatory BM microenvironment that promotes mutant HSPC growth, inhibits hematopoiesis in healthy HSPCs, and predisposes mutant HSPCs to a certain spectrum of secondary “hits” that lead to AML transformation [6]. Ineffective hematopoiesis-associated cytopenia and an increased risk of AML transformation are the two of the primary issues facing MDS patients. Eliminating mutant HSPC clones is essential to curing MDS, but most medications used to eliminate them suffer from a lack of specificity and relatively high toxicity, especially in older patients. As a result, current therapies for MDS are little more than palliative, with a 5 year survival rate of ~ 37% [1].

MDS develops from an accumulation of genomic lesions in HSCs. Many cytogenetic abnormalities and gene mutations have been identified in MDS patients (Table 1). At least one such molecular abnormality can be detected in the blood of approximately 94% of MDS patients. Among them, 53% of patients have gene mutations only, 4% have cytogenetic alterations only, and 37% have both [7]. Although most patients have more than one genetic abnormality, mutations in *SF3B1* and *TET2*, as well as del(5q), are enriched in patients with only one driver event. The presence of three or more mutations is associated with an inferior overall survival (OS) [8]. In addition, the evolution of genomic lesions in MDS patients is not always linear; thus, more than one mutant clone of hematopoietic cells can be detected in some MDS cases [9, 10].

**Table 1:** Common cytogenetic abnormalities and mutant genes in MDS [11]

*Common cytogenetic abnormalities*
− 7/del(7q) and − 5/del(5q), + 8, dup(1q), del(20q), del(11q), del(12p)/t(12p), del(17p)/iso(17q), del(18q), + 21q, del(13q), + der(1;7)(q10;p10)
*Common somatic gene mutations*
RNA splicing: *SF3B1*, *SRSF2*, *U2AF1/2*, *ZRSR2, LUC7L2* Epigenetic/chromatin modifiers: *TET2*, *DNMT3A*, *IDH1/2*, *PHF6*, ASXL1, *EZH2*, *BCOR*, *bCORL*, *MLL* DNA damage response: *TP53*, *PPM1D* Transcription factors: *RUNX1*, *ETV6*, *GATA2*, *CUX1* Signal transduction: *NRAS*, *KRAS*, *JAK2*, *PTEN*, *CBL*, *NF1*, *PTPN11*, *MPL*, *FLT3* Cohesin complexes: *STAG2*, *MAU2*, *SMC3*, *RAD21* and *SMC1A*

Anemia is the most common symptom in MDS, presenting in > 80% of cases. In some patients with mild anemia, their disease is stable with near-normal life expectancy, requiring only supportive treatments. However, ⁓50% of patients with hemoglobin (Hb) < 10 g/dL symptomatic anemia present with dizziness, fatigue, diminished cardiopulmonary function, an increased tendency to cardiopulmonary failure, and cognitive decline, eventually leading them to becoming red blood cell (RBC) transfusion dependent (TD) [12, 13]. Thrombocytopenia occurs in 40–65% of MDS patients, increasing the risk of bleeding complications (accounting for ~ 10% of all deaths); neutropenia and neutrophil dysfunction lead to severe infections (accounting for 18% of all deaths) [14, 15]. Additionally, 30–40% of MDS patients go on to develop AML and die within 4–6 months after transformation [16]. Thus, more intensive treatments are necessary to restore blood cell counts and prevent AML transformation in these MDS cases.

Due to the heterogeneic features of MDS and the potent toxicities associated with many of the medications, current therapeutic decision-making for MDS is primarily determined based on disease classification and risk scores. WHO and ICC diagnostic classification systems (Supplementary Table 1), as well as IPSS and WPSS scoring systems, have been developed to predict AML transformation, disease progression, and outcome. During the last 10 years, the revised IPSS (IPSS-R, https://www.mds-foundation.org/ipss-r-calculator/) has commonly been used in most medical centers. Based on the hematologic parameters *(e.g.*, anemia, thrombocytopenia, and neutropenia in PB; myeloblast percentage in BM) and cytogenetic abnormalities, IPSS-R classifies newly-diagnosed and untreated MDS patients into 5 risk groups (very low, low, intermediate, high, and very high) [17]. The intermediate risk (IR) group can be further divided into IR-1 and 2. Based on IPSS-R, patients in clinics are generally divided into either a low-risk group (LR, IPSS-R ≤ 3.5, including very LR, LR, and IR-1) or a high-risk group (HR, IPSS-R > 3.5, including IR-2, HR, or very-HR). The standard of care for LR-MDS is focused on reducing cytopenia-related symptoms, reducing the number of transfusions, and minimizing morbidity in order to improve the quality of life and extend the lifespan. The treatment goals for HR-MDS aim to delay AML transformation, prolong survival, and improve the quality of life through improvement of PB cell counts [18]. More recently, IPSS-Molecular (IPSS-M, https://mds-risk-model.com/) was developed by incorporating the values of 31 somatic gene mutations into the IPSS-R that refines the classification of MDS into 6 risk groups. Compared with the IPSS-R, IPSS-M improves prognostic accuracy across all long-term clinical end points (*e.g*. leukemia-free survival, leukemic transformation, and overall survival) in both primary and secondary therapy-related MDS [7, 19–21], suggesting IPSS-M is a better system to risk-stratify MDS patients for optimized therapeutic decision-making.

## Standard treatments for LR-MDS

In clinical studies, Hb levels < 10 g/dL are usually accepted as an indicator for the initiation of treatment, and hematopoietic improvement (HI, ≥ 1.5 g/dL increase of Hb) and transfusion independence (TI) for at least 8 weeks are commonly used as responses and endpoints for treatments. However, due to the different symptomatic responses of patients to anemia, particularly in cases of cardiovascular comorbidity, anemia-associated symptoms are commonly used as reasons to initiate intervention. Blood cell transfusion is a common supportive therapy for cytopenia, which can lead to complications like circulatory overload, lung injury, iron overload (IOL), and disease transmission, causing inferior progression-free survival [22, 23]. After receiving infusions of more than 20–50 units of RBCs, almost all patients become IOL. Excess iron not only causes tissue damage, leading to organ dysfunction (including chronic liver disease, cirrhosis, heart failure, arrythmias, and diabetes) but also further impairing hematopoiesis [24–26]. Therefore, several treatments have been developed to combat these anemia-associated symptoms in order to reduce TD.

### Treatments for non-del(5q) MDS patients with symptomatic anemia

In non-del(5q) MDS patients with symptomatic anemia, erythropoiesis-stimulating agents (ESAs) such as recombinant humanized erythropoietin (EPO, epoetin-α) or the longer-acting EPO (darbepoetin-α, which has not been approved outside the USA) are the front-line treatments that improve RBC counts in 20–40% of patients for 8–23 months. Approximately 70% of ESA responders eventually return to TD [27–30]. No clear differences were observed between treatment with epoetin-α or darbepoetin-α when optimal dose/schedule administrations were used [27, 29, 31]. ESAs target the early stages of erythropoiesis by inhibiting apoptosis, stimulating proliferation of erythroid progenitors, and regulating terminal maturation for EPO-responsive erythroid precursors without significantly affecting the size of the disease clones [32, 33]. ESA treatment is typically limited to patients with < 500 IU/L endogenous serum EPO levels (sEPO), with better responses in those with sEPO < 200 IU/L, low to moderate TD (< 4 units/8 weeks), and lower mutational burden (LMB, ≤ 2 somatic mutations) [28, 34]. Mutations in *STAG2, GNAS*, and *SRSF2* were associated with a worse OS [35]. Patients with *SF3B1* mutations responded equivalently or even better to ESAs than those without *SF3B1* mutations, albeit for a shorter median duration [36]. Addition of G-CSF is recommended, which might have an additive effect to ESA-stimulated erythroid responses; however there is concern that it may increase the risk for transformation to AML in patients with leukemic mutations [27, 37, 38]. Increased Hb levels and decreased TD are associated with improved quality of life and overall survival (OS) [31]. Initiation of ESA treatment before the onset of permanent TD might be beneficial in significantly prolonging the time to first transfusion [39]. Treatment failure in patients with an initial response to ESAs is associated with increasing HSC clonal dominance and not disease progression. Novel medications are needed for such patients because second-line treatments, including hypomethylating agents (HMAs; not approved outside the USA) and lenalidomide (LEN), promote HI and TI in a subset of patients without improving their OS significantly [40, 41]. For patients with multiple cytopenias, shorter dosing schedules of HMAs like azacitidine (AZA), decitabine (DEC), and oral decitabine-cedazuridine are recommended, as they provide similar response rates to HR disease with a median response of 12–18 months [42–46].

### LEN for MDS patients with del(5q)

Patients with del(5q) MDS are prone to macrocytic anemia, increased platelet counts in PB and hypolobulated megakaryocytes in the BM [47]. Patients with del(5q) MDS usually have high sEPO levels. Approximately 80% of these patients do not respond to treatment with ESA [48]. LEN was approved as a first-line treatment for patients with del(5q). LEN is an immunomodulatory derivative of thalidomide which induces ubiquitination and degradation of casein kinase Iα (CK1α) and CDC25C to exploit a defect in ribosomal protein function [49]. Both CK1α and CDC25C are encoded by genes within the commonly deleted region of del(5q) MDS. The haploinsufficient expression of CK1α and CDC25C makes del(5q) MDS HSPCs hypersensitive to LEN-induced p53-dependent apoptosis and CDC25C and PP2A-related cell cycle arrest [50]. LEN (10 mg daily) reduced TD in 65%–70% of LR del(5q) cases and induced a complete response (CR) in 30%–40% of cases, with a median duration of response of more than 2 years without increased incidence of AML transformation. [51–54]. MDS‐stem cells (SCs) are resistant to LEN and can cause relapsed disease or progression in most patients, especially in patients with *TP53* mutations [55–58]. Therefore, LEN is not recommended for patients with *TP53* mutations, specifically those with biallelic or > 20% monoallelic mutations [56]. In addition, more than 70% of treated patients develop grade 3 or 4 neutropenia, and more than 1/3 of patients develop thrombocytopenia [59]. A recent study suggests that early low-dose LEN intervention (5 mg) significantly extends TI survival and improves quality of life in 69.8% of patients with TI del(5q)-MDS, suggesting that early low-dose LEN treatment is beneficial [60].

For ESA-failed non-del(5q) MDS patients, LEN has been used as a rescue therapy, inducing a 25–35% overall response rate (ORR) and > 8 weeks RBC-TI in 20% of patients. A combination of LEN + ESA shows moderate additive effects on erythropoietic enhancement (up to 40%) and TI (up to 25%) and can be used as a salvage therapy [41, 61]. Predictive factors for a response to LEN in non-del (5q) MDS include a low percentage of BM lymphocytes and progenitor B-cells, LMB and the absence of ring-sideroblasts (RS) and *SF3B1* mutations [62]. Mutations in *U2AF1* and DEAD-box RNA helicase genes (*DDX41*, *DDX54*, and *DHX29*) were significantly associated with failure to respond to LEN [63]. The presence of a mutation(s) in any of five genes (*ASXL1*, *ETV6*, *EZH2*, *RUNX1*, *TP53*) was associated with a significantly shorter median OS in LEN-treated patients [41].

### Immune-suppressive therapies (IST) for patients with BM hypocellularity

A subset (10–15%) of MDS patients with concomitant autoimmune diseases displays a BM hypocellularity of ≤ 30% [64]. Expansion of CD3^+^CD4^−^CD8^+^CD16^±^CD56^−^CD57^+^ cytotoxic T-large granular lymphocytes or CD3^−^CD16^bright^CD56^dim/neg^ NK cells was observed in these patients [64]. In a meta-analysis of published data in multiple clinical trials, Stahl et al*.* analyzed the clinical responses of 207 and 570 LR-MDS patients who received anti-thymocyte globulin (ATG)-based IST (including ATG + cyclosporin A, tacrolimus or etanercept). ATG-based IST induced ORR in 42.5–48.8% of patients, including 11.2–12.5% of patients who achieved CR and 30–33.4% who achieved RBC-TI [65]. Median OS was 47.4 months. However, the progression rate to AML was 8.6% per patient year [65, 66]. Currently, IST is only recommended to treat young patients with hypocellular and hypoplastic BM with LMB in genes associated with poor prognosis [18, 65].

### Iron chelation therapy (ICT) for TD patients with IOL

ICT has been designed to remove excess iron from the body by binding to iron and helping to get rid of it through urine and/or feces. Sufficient evidence suggests that ICT has a significant clinical benefit for LR-MDS patients with IOL [67, 68]. ICT not only improves the function of vital organs (*e.g.*, liver and heart) but also induces hematopoietic improvement in erythroid cells (10–40%), neutrophils (7–20%), and platelets (10–30%), as well as lowers the risk of AML progression in some cases. Reduction of RBC transfusion was observed in many of the responders, and ⁓10% of patients achieve RBC-TI [67, 68]. Compared to patients without ICT, patients receiving ICT experience a longer time to first infection and reduced risk of complications with superior OS, cardiac event-free survival, and possibly leukemia-free survival [68–70]. In addition, IOL has a significant negative impact on the success of allo-HSCT. For HR-MDS patients with IOL and receiving allo-HSCT, ICT reduces tissue damage and improves HSC engraftment and hematopoietic recovery, leading to a lower risk of complications and improved relapse-free survival [71, 72]. Currently, ICT is recommended for LR-MDS patients displaying IOL (serum ferritin (SF) > 1000 ng/mL) with > 2 years of projected life expectancy as well as for HR-MDS patients with IOL, and it is being considered for allo-HSCT [73]. However, for patients with high transfusion requirements, preexisting organ dysfunction, and indicators of oxidative stress, ICT can be used even in a mild IOL (SF < 1000 ng/mL). Two oral ICTs, deferasirox (DFX) and deferiprone (DFP), as well as a single injection of ICT deferoxamine (DFO) have been approved for such purposes. ICT usually needs continuous administration, with dose reduction when SF reaches < 1000 ng/mL, and withdrawal until SF returns to normal (< 500 ng/mL). However, the SF concentration indirectly estimates iron burden and can be influenced by many other conditions (inflammation, infections, and liver disease). Its decrease is not always associated with chelation response. Thus, better biomarkers for IOL, such as transferrin saturation [73], non-transferrin-bound iron, labile plasma iron [74, 75], or magnetic resonance imaging for cardiac iron are needed to instruct ICT in the clinical setting [75]. The common adverse events (AEs) of ICT are primarily gastrointestinal, including constipation, diarrhea, nausea, and vomiting which normally stop once the body adjusts to the iron. Continuous DFO injections cause reactions, pain, infections, and bleeding at injection sites. Renal defects and neutropenia/agranulocytosis have been reported in patients treated with DFX and DFP, respectively. Thus, DFX should be avoided for patients with renal impairment, and DFP should be avoided with patients undergoing HMA treatment with neutropenia.

## Standard treatments for HR-MDS

### Allo-HSCT

Allo-HSCT is the only curative option for HR-MDS [76]. Allo-HSCT induced a 50% 3-year OS rate in HMA-naïve patients, a significant improvement compared to 26.6% in the HMA-treatment arm [77]. For HMA treated patients, event-free survival rates of 3 years were 34% in the allo-HSCT arm and 0% in the AZA arm [78]. However, HSCT is a highly toxic therapy (age-adjusted therapy-related mortality by 2 years is ⁓20%) [79]. Pre-existing conditions, such as heart and chronic kidney diseases, are commonly associated with high therapy-related mortality, which limits allo-HSCT utility [80]. With reduced intensity conditioning regimens, patients up to 75 years of age are still eligible for allo-HSCT [77]. Clinically, decisions for HSCT are primarily made based on disease risk scores. For HR-MDS, immediate HSCT is recommended (optimal timing is within 5 months of diagnosis), regardless of age. In addition, due to the poor prognosis of *TP53*-mutant patients (regardless of age and risk score) and patients ≥ 61 years old with LR to IR-1 MDS, immediate allo-HSCT is also recommended. However, if allo-HSCT is not feasible in the short term, HMA treatment could be used as a bridge. For the remaining LR-MDS patients, a delayed approach to allo-HSCT is recommended [81–83].

### HMA treatment

For allo-HSCT non-eligible patients, HMAs such as DEC (has not been approved in Europe) and AZA have been recommended as first-line treatment [84–86]. HMAs function by competing with nucleotide analog deoxycytidines to incorporate into newly-synthesized DNA strands during S-phase. Low dose HMA treatment that is sufficient to deplete S-phase–dependent DNA methyltransferase 1 (DNMT1) triggers the differentiation of committed mutant HSPCs without affecting the self-renewal of healthy HSCs [87, 88]. In addition, low dose HMAs also trigger type 1 interferon production and RNase L–mediated cell death by reactivating endogenous retroviruses [89–93]. However, high dose HMAs induce cell-cycle dependent DNA damage and p53-dependent apoptosis, which cause off-target AEs in normal hematopoiesis [94, 95]. In the clinic, standard HMA regimens are ≥ 6 cycles of intravenous or subcutaneous AZA (50–75 mg/m^2^/d. for 5–7 days) or DEC (20 mg/m^2^/d. for 3–5 days) every 4 weeks. This regimen induced ORR/clinical improvements in > 50% of patients and a CR in < 20% of patients [84, 96, 97]. Relapses and drug resistance are inevitable due to the persistence of MDS-SCs from the same major clone present at diagnosis, suggesting epigenetic and/or metabolic reprogramming mechanisms drive the processes of clonal evolution [98]. Most responders relapse within 2 years, and their OS is approximately 18.6 months (range, 15.3–21.9 months) [99]. However in real-world analyses, the OS was 12–13 months, significantly lower than that observed in clinical trials [96, 100, 101]. More importantly, primary HMA response does not always predict better OS due to secondary resistance. CR and marrow CR are often associated with a reduction of mutant clones which predict a long-term outcome, specifically in *TP53*-mutant cases after subsequently receiving allo-HSCT [102–105]. Although TP53 is not required for HMA-induced termination of mutant HSPCs, *TP53* inactivation upregulates the endogenous de novo pyrimidine synthesis pathway that directly antagonizes the DNMT1-depleting nucleotides [106], explaining why TP53 abnormalities are still an adverse prognostic biomarker in HMA-treated patients [107]. The most frequent grade 3/4 hematologic AEs are myelosuppression-associated neutropenia, anemia, and thrombocytopenia. The prognosis for HMA non-responders (primary resistant and acquired refractory) is very poor, with a median OS between 4.3–5.6 months and a 1-year OS of 28% and 2-year OS of 15% [108, 109]. Although allo-HSCT is still effective in a small proportion of cases, novel medications are urgently needed.

Cytidine deaminase (CDA) is a master regulator of the pyrimidine metabolic pathway which catalyzes the hydrolytic cleavage of HMAs and is highly expressed in the intestines and liver [110]. CDA is up-regulated in many HMA-resistant cases [111]. CDA inhibitors such as tetrahydrouridine and cedazuridin (CED) have been evaluated for reversing HMA resistance in preclinical studies, but whether they can reverse HMA resistance in patients needs to be determined. INQOVI is an orally bioavailable DEC, which is a mixture of 35 mg DEC and 100 mg CED [42]. As CDA is highly expressed in the intestine and liver, INQOVI overcomes the limitation of rapid metabolism of DEC by CDA in these organs, making it possible to treat patients without injections. In both phase II and phase III multicenter, randomized clinical trials, INQOVI induced TI in ⁓49% of patients and CR in 18–20% of patients, with a 7.5–8.7 month median duration of CR that were comparable to DEC treatment [112, 113]. However, oral administration of HMAs might increase the rate of gastrointestinal AEs compared to injections. Based on the results from these studies, the FDA approved INQOVI for adult patients with IR-1 and HR-MDS in July, 2020 [112].

## Recent advances in MDS research and treatments

The past several years have seen significant progress in the treatment of MDS, including two FDA-approved drugs for LR-MDS and targeted therapies that have been evaluated for genetic mutations. Many new drugs are still under evaluation in clinical trials with or without an HMA combination.

### Luspatercept targets TGFβ-SMAD signaling for treatment of LR-MDS with symptomatic anemia

TGFβ and its family members Activin A, TGFβ1, BMP2/4/9, and GDF11/15 have been found to play critical roles in the regulation of erythropoiesis [114–117]. Sustained TGF-β signal activation was detected in MDS, as demonstrated by increased p-SMAD2 and a reduction in inhibitory SMAD7 [118]. GDF11 and GDF15 are upregulated in MDS patients, specifically in patients with refractory anemia and ring-sideroblasts (RA-RS) that inhibit the late-stages of erythroid differentiation [119]. Several TGF ligand traps have been developed and are being evaluated in clinical treatment trials for MDS, including luspatercept, sotatercept, and KER-050 [120, 121]. Luspatercept is a modified Activin type IIB receptor (Act-RIIB)-fusion protein used to inhibit canonical TGFβ-SMAD signaling, inducing later stage maturation of erythroblasts [122, 123]. The phase II multicenter, open‐label PACE trial approved a 1–1.75 mg/kg once every 3 weeks subcutaneous (s.c.) injection of luspatercept as safe, which led to long-term clinical efficacy in a proportion of patients [124]. In the MEDALIST trial, luspatercept treatment of patients with TD, LR to IR-1 MDS-RS resulted in TI for at least 8 weeks during the first 24 weeks of treatment in 58/153 (37.9%) of patients, which was significantly higher compared to 10/76 (13.2%) in patients who had received placebo [121]. Patients who achieved 8-weeks of TI were associated with improved OS [125]. This promising result led the FDA, on April 3, 2020, to approve luspatercept as a first-line treatment for patients with MDS-RS and MDS-RS-thrombocytosis who have failed to responded to or are ineligible for ESA treatment. As with ESAs, luspatercept is recommended for patients with sEPO levels < 500 IU/L. More recently, the COMMANDS trial demonstrated that among ESA-naïve patients, luspatercept treatment led to HI and TI for at least 12 weeks in 109/178 (74%) and 98/178 (67%) of patients, respectively, which were significantly better than HI in 79/178 (51%) and TI in 71/178 (46%) of ESA‐treated patients. Amongst patients that achieved a 12-week TI, the median duration of TI was 127 weeks in the luspatercept arm, which was significantly longer than the 77 weeks in the ESA arm. The response rate of luspatercept treatment was higher than that of ESA in RS-positive patients; however in RS‐negative patients, the response rates were comparable between the two treatment groups [34]. Similar to ESA treatment, luspatercept did not inhibit the mutant clone [126]. However, in contrast to ESA treatment, the efficacy of luspatercept treatment was less influenced by mutational burden [34]. The AEs occurring among luspatercept-treated patients were almost comparable to those among ESA-treated patients [127]. This promising result led to FDA approval for luspatercept as a first-line treatment for adults with LR-MDS and TD anemia with sEPO levels ≤ 500 U/L on August 28, 2023. Thus, it is time to promote luspatercept to replace ESA as a standard first-line treatment for RS-positive patients, while for RS-negative patients, future study needs to determine whether sequential or combination treatment with luspatercept and ESA can improve the long-term treatment efficacy [128]. In addition, a study is ongoing to evaluate the efficacy of luspatercept versus ESA in the first-line setting for non-BRC-TD and ESA-naïve LR-MDS patients.

### Targeting telomerase for MDS treatment

Although MDS HSPCs have shorter telomeres than healthy controls, they have higher telomerase activity, potentially due to a feedback mechanism. Higher levels of telomerase prevent programmed cell death (PCD) in mutant HSPCs, predicting disease progression and poor prognosis [129]. Imetelstat is a 13-mer oligonucleotide with a lipid moiety that specifically binds the RNA template of human telomerase and acts as a potent, competitive inhibitor of telomerase’s enzymatic activity [130]. Based on data from the phase 3 IMerge trial, imetelstat selectively induces apoptosis in the malignant HSPCs of MDS patients. Imetelstat reduced cytogenetically abnormal clones and mutational allele burden as demonstrated by a reduction of variant allele frequency (VAF) in the mutant genes, resulting in RBC-TI for > 8 weeks and > 24 weeks in 40% and 28% of patients with a median TI duration of 65 weeks regardless of transfusion burden (TB) or RS status [131]. TI was durable with imetelstat, with a median duration of RBC-TI of 51.6 weeks and with a median rise in Hb of 3.6 g/dL. Most importantly, all patients studied in this trial were ESA-relapsed, ESA-refractory, or ESA-ineligible (sEPO > 500 IU/L) LR-MDS. Some prior LEN and luspatercept treatment patients are still responding to imetelstat treatment [132]. Therefore, imetelstat can be used as a first line treatment for non-del(5q) LR-MDS patients with sEPO > 500 IU/L and/or high TB (HTB, ≥ 4 U/8 weeks) and category 1 s-line therapy for patients that are relapsed/refractory or ineligible for ESAs, as approved by the FDA on June 6, 2024. However, imetelstat treatment commonly caused significant AEs such as grade 3–4 neutropenia and thrombocytopenia in 68% and 62% of patients, and 18% of patients needed platelet transfusion support during treatment. Thus, further optimization of the dose/schedule of imetelstat, together with improved supportive therapy, is needed.

### Targeting mutant genes for MDS treatment

*IDH1* and *IDH2* mutations are rare in MDS (~ 5%) [133] but can be targeted by specific inhibitors. A multicenter phase I study [134, 135] demonstrated that treatment with the IDH1 inhibitor ivosidenib induced CR + partial remission (PR) in 39% (7/18) and CR + PR + marrow CR in 83.3% of relapsed and refractory (R/R)-MDS patients with *IDH1* mutations. The duration of CR ranged from 1.9 to 80.8 months. Among the nine patients who were TD, six (67%) no longer required transfusions [136]. On October 24, 2023, the FDA approved ivosidenib for the treatment of adult patients with R/R-MDS with an *IDH1* mutation. In a multicenter, investigator-initiated phase 2 clinical trial, a total 55 patients with *IDH2*-mutant MDS were recruited [137]. Twenty-seven newly-diagnosed patients were treated with the combination of enasidenib (an IDH2 inhibitor) + AZA, and 23 with prior HMA treatment were treated with enasidenib monotherapy. In the combination arm, the ORR was 74% and the median OS was 26 months. In the enasidenib monotherapy cohort after HMA failure, ORR and CR were 35% and 22%, respectively; the median OS was 20 months. This study suggests that enasidenib is an effective treatment option for *IDH2-*mutant MDS, both in combination with AZA for treatment-naïve HR-MDS and as a single agent after prior HMA therapy. [137].

### ***CPX-351 is intended for use in HR MDS and CMML*** [138, 139]

Standard chemotherapy for AML did not show any benefit for HR-MDS patients compared to HMA. CPX-351 is a liposomal encapsulated form of cytarabine and daunorubicin (5:1 ratio) that showed greater efficacy in treating secondary AML and reduced non-hematopoietic AEs compared to the classic 7 + 3 regimen [140]. It was used to treat adults with newly-diagnosed therapy-related AML or AML with myelodysplastic type changes (AML-MRC). Recently, a multicenter, single-arm, phase 2 study of 31 newly-diagnosed MDS with excess blasts (MDS-EB) patients demonstrated that CPX-351 induced an ORR of 87% with a 12-month DFS rate of 67.7% and an OS of 80.6% [138]. Almost all patients subsequently proceeded to allo-HSCT. This study suggests that CPX-351 is active and safe in patients with HR-MDS and CMML, allowing for a bridge to allogenic HSCT in most patients. Randomized comparative studies are needed to determine whether CPX-351 is more effective than HMA in HR-MDS, with acceptable levels of AEs.

## Recommended treatment strategy for MDS patients

**Fig. 1:**
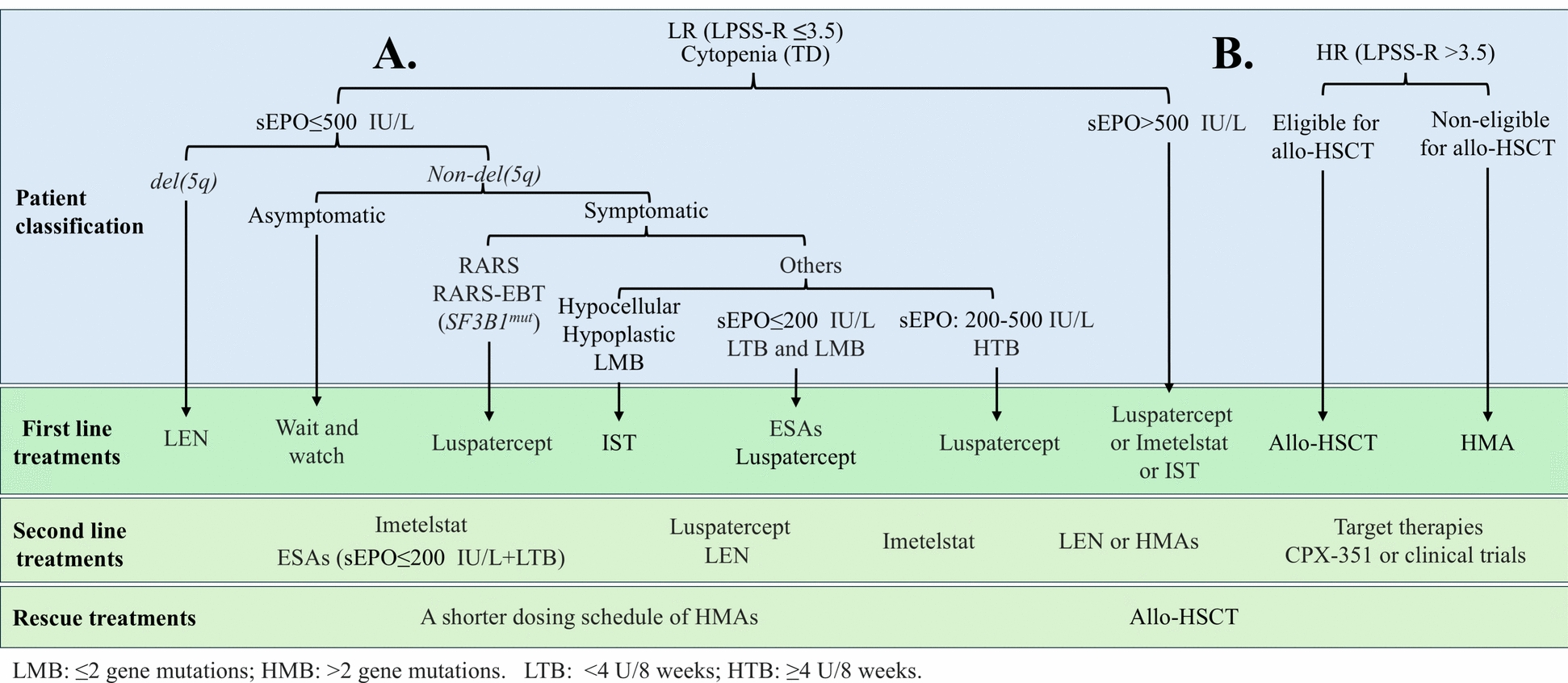
Current treatments for MDS patients. Patients are classified based on LPSS diagnostic criteria. Based on the classification into (**A**) LR or (**B**) HR in addition to other factors, different treatment strategies are employed. LR: low-risk; HR: high-risk; TD: transfusion dependency; RARS: refractory anemia with ring sideroblasts; LEN: lenalidomide; IST: immune-suppressive therapies; ESAs: erythropoietic stimulating agents; allo-HSCT: allogeneic HSC transplantation; HMA: hypomethylating agents

With all these advances during the past several years, for del(5q) MDS, LEN is still the first choice, whereas, for non-del(5q) MDS, in addition to the IPSS scores RS status, sEPO levels, mutational burden (MB), and transfusion burden (TB) are also critical for treatment decision making. For RS-positive LR-MDS RBC-TD patients, luspatercept should be used as the frontline treatment option given the higher response rates and longer response duration times when compared to ESAs. However, for RS-negative LR-MDS, if sEPO levels are ≤ 200 U/L, MB ≤ 2, and TB < 4 U/8 weeks, both ESAs and luspatercept can be used as front-line options given their comparable response rates. Two recent studies in mice demonstrated that inhibition of either TGFβ1 or BMP9 induces the production of erythroid progenitors and promotes the EPO-dependent production of RBCs [115, 117]. Thus, besides the financial cost, future study needs to determine which treatment gives better long-term OS, which one should be given first if administrated sequentially, and the potential benefit of combination treatment. For RS-negative LR-MDS with sEPO levels of 200–500 IU/L, if TB is < 4 U/8 weeks, luspatercept should be considered as frontline treatment; however, if TB ≥ 4 U/8 weeks, imetelstat should be considered the frontline treatment. For patients with sEPO levels > 500 IU/L, either luspatercept or imetelstat can be selected. In addition, many patients with sEPO > 500 IU/L most likely will respond to IST with eltrombopag treatment. After the failure of front-line treatments, the second line treatment will be alternating the use of LEN, luspatercept, or imetelstat (the best sequencing of these therapies needs to be determined), while HMAs and allo-HSCT will be used as rescue treatments. However, for multilineage cytopenic patients, a shorter dosing schedule of HMAs can be considered as first line treatment (Fig. 1A**)**. The recommended treatment regimen for each medication is summarized in Table 2. For patients with HR-MDS, allo-HSCT should be considered as the first choice as long as they are eligible and have proper HLA-matched donors. However, for non-eligible patients, HMAs are currently the only option. For HMA treatment failures, targeted therapies, CPX-351, or clinical trials should be considered (Fig. 1B).

**Table 2:** Recommended treatment regimens for FDA approved medications

Treatments	Medications	Recommended regimens
ESAs	Epoetin-α [29] Darbepoetin-α [27]	Epoetin-α is recommended to be administrated subcutaneously. The initial dose is 30,000–40,000 units (450 units/kg body weight) once a week for 8 weeks. If there is no response, the dose is increased to 60,000–80,000 units (1050 units/kg body weight) once a week for another 8–12 weeks. For all non-responders, G-CSF can be added with a starting dose of 300 µg once a week which can be increased to 300 µg three times per week for another 8-weeks. However, the dosing regimen should be tailored to individual patients according to need and response, as well as body size The starting dose for darbepoetin is 300 µg once every 2 weeks or 150–240 µg once every week. This can be increased after 8 weeks in non-responders to a maximum of 300 µg per week for a further 8 weeks. For all non-responders, G-CSF can be added for another 8–12 weeks For both epoetin-α and darbepoetin-α treatments, if Hb levels increase to > 12 g/dL, or there is a rapid increase in Hb levels (> 2 g/dL over any 4-week period), treatment will be interrupted. Detailed disease assessments are carried out every 24 weeks. Treatment continues until disease progression, lack of clinical benefit, unacceptable toxicity, or consent withdrawal
Immuno-modulator	lenalidomide (LEN) [53, 84]	10 mg daily for 21 days repeated every 28 days continuous until it no longer works, or unacceptable side effects occur
ATG-based IST	ATG + cyclosporin A (CsA) [65] ATG + Etanercept	ATG at 40 mg/kg/day for 4 consecutive days, followed by CsA 5–12 mg/Kg/day for 6 months ATG at 40 mg/kg/day for 4 consecutive days, followed by subcutaneous etanercept at 25 mg twice a week for 2 weeks, every month for 4 months
ICT	Deferasirox (DFX) [67] Deferiprone (DFP) Deferoxamine (DFO)	Oral 20 mg/kg/day (dispersible tablets) or 14 mg/kg/day (Film-coated tablets) of DFX Oral 75 mg/kg of DFP 3 times daily Subcutaneous injection of 20–60 mg/kg/day over 8–24 h. IV: 20–40 mg/kg/day (children) or 40–50 mg/kg/day (adults) over 8–12 h ICT requires continuous administration, with dose reduction when SF reaches < 1000 ng/mL, and withdrawal until SF returns to normal (< 500 ng/mL)
HMAs	Azacitidine (AZA) [141] Decitabine (DEC) [142] INQOVI [112, 113]	AZA 75 mg/m^2^ sc. for 7 consecutive days, repeated at 28-day intervals for 6 cycles. Responding patients should continue while their response is maintained DEC 20 mg/m^2^ for five days every 4 weeks for 6 cycles. Responding patients should continue while their response is maintained INQOVI tablet (35 mg decitabine + 100 mg cedazuridine) orally once daily on days 1–5 of each 28-day cycle until disease progression or unacceptable toxicity
TGF ligand traps	Luspatercept [34, 125, 127]	1.0 mg/kg subcutaneously, once every 3 weeks. This can be increased after 7 weeks in non-responders to 1.33 mg/kg and then can be further titrated up to 1.75 mg/kg, once every 3 weeks. Detailed disease assessments are carried out on week 24 and every 24 weeks thereafter. Treatment continues until disease progression, lack of clinical benefit, unacceptable toxicity, or consent withdrawal
Telomerase inhibitor	Imetelstat [131, 132]	Intravenous infusion of 7.5 mg/kg over 2 h every 4 weeks until disease progression or unacceptable toxicity
IDH1 inhibitor	Ivosidenib [136]	500 mg daily orally (250 mg once daily when combined with CYP3A4 inhibitor) continuous until disease progression, unacceptable toxicity, or hematopoietic stem cell transplantation

## The major challenges in MDS research and treatments, as well as potential solutions

**Fig. 2:**
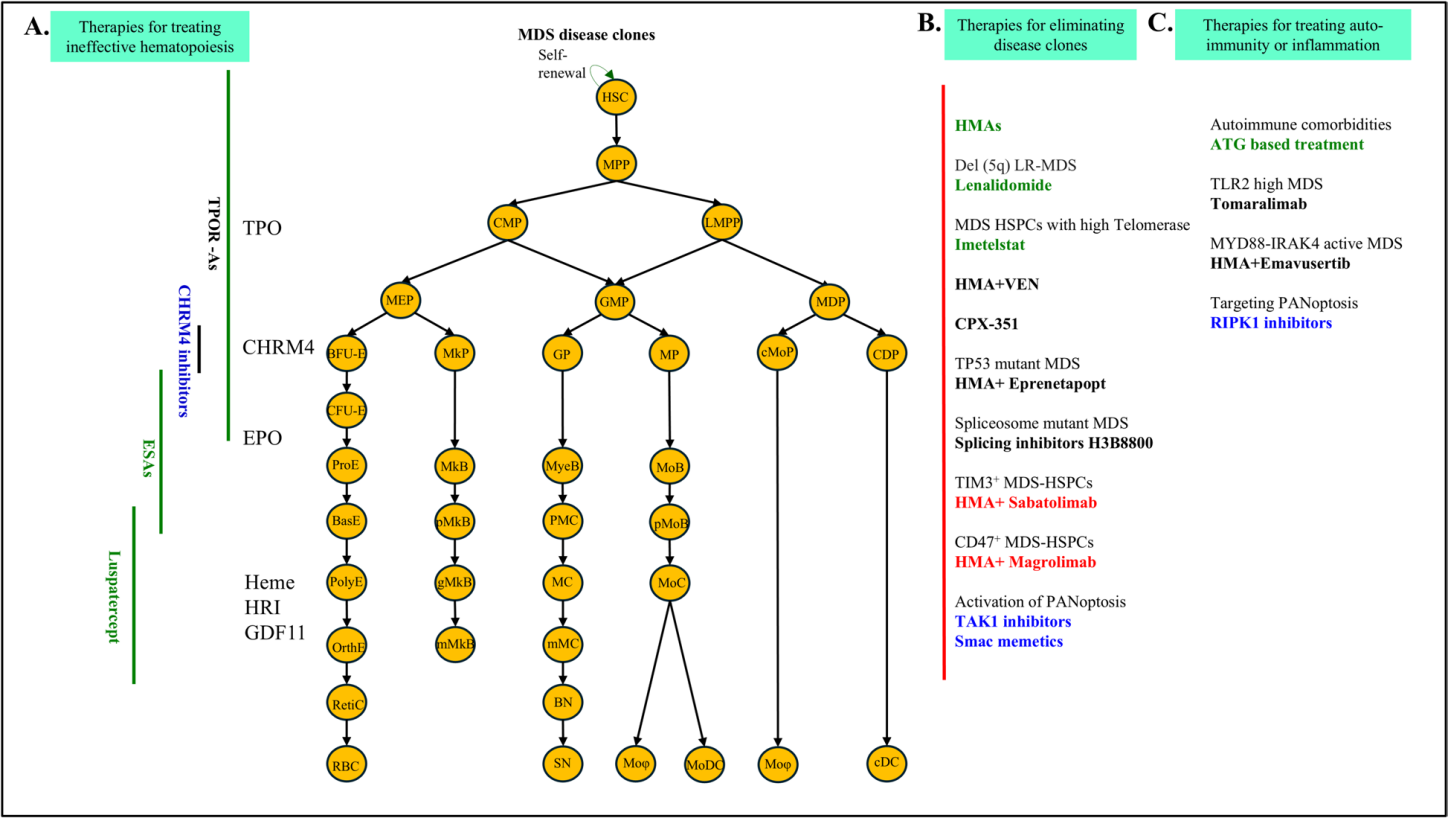
Diagram of MDS treatments and mechanisms. Based on patients’ clinical symptoms and IPSS-R scores, the treatment strategies for MDS patients include: **A** Improving blood cell counts to reduce cytopenia-related symptoms and morbidity by stimulating erythropoiesis and thrombopoiesis. **B** Targeting disease clones to delay AML transformation and prolong survival. **C** Targeting the innate immune system for MDS patients with overactive TLR signaling, targeting autoimmune signaling for MDS patients with hypocellular BM, and inhibiting RIPK1 for MDS with RIPK1-dependent cell death. The agents shown in green font are FDA-approved medications. The agents in black font are still in clinical trial evaluations, whereas the clinical trials for the agents in red font were terminated due to failure to meet their primary endpoints. The agents in blue font are still in pre-clinical evaluation

Although HMAs induce apoptosis of MDS-HPCs in sensitive patients, MDS-SCs are resistant. To improve HMA efficiency, many HMA-based combination therapies have been evaluated in early clinical trials. Several of these combined agents seem to target MDS-SCs but are associated with increased toxicity and treatment-related death (Fig. 2). Among them, only HMA + venetoclax (VEN, a Bcl-2 inhibitor) [143], HMA + sabatolimab (SAB, an anti-TIM-3 antibody) [144], HMA + magrolimab (MAG, an anti-CD47 antibody) [145] and HMA + Eprenetapopt (EPR, a TP53 activator) have shown promising results during early clinical trials. The combinations of an HMA with inhibitors of any epigenetic modifier (including HDAC and NEDD8) have not demonstrated a survival benefit when compared to monotherapy, likely due to greater toxicity and dosing modifications that limit drug exposure in patients treated with drug combinations.

### The balance between benefits and toxicity

#### Targeting BCL2 for MDS treatment

In AML, HMAs induce the expression of pro-apoptotic proteins and repress the expression of the pro-survival protein MCL1. However, most AML blasts, including leukemia stem cells (LSCs), express high levels of another pro-survival protein, BCL2, thus compromising the anti-AML effect of HMA. VEN inhibits BCL2 and impairs mitochondrial processes for de novo pyrimidine synthesis, thus restoring AML cell sensitivity to HMAs [146]. HMA + VEN induces > 60% ORR in older, previously untreated AML patients, with the majority of these patients achieving CR [147]. Consequently, HMA + VEN has been approved by the FDA as a first-line treatment for AML patients ≥ 65 years old. MDS HSPCs also express high levels of BCL2. HMA + VEN has been evaluated in phase I and phase I/II clinical trials for the treatment of newly-diagnosed HR-MDS, showing ORR in 74% and CR/CRi in 66% of patients [143, 148]. HMA + VEN has also been shown to provide a moderate benefit in relapsed MDS post-allo-HSCT and 44% ORR in patients after HMA failure [143, 149]. However, significant myelosuppression in > 50% of patients characterized by severe febrile neutropenia, thrombocytopenia, and anemia-related higher AEs and increased treatment-related death limit its use, as it does not significantly extend the OS of patients compared to HMA-only treatment. Nevertheless, OS is significantly longer in HMA + VEN-treated patients followed by allo-HSCT, suggesting its potential as a bridge to transplantation [143]. More recently, Garcia et al. demonstrated that short-scheduled low dose VEN (400 mg for 14 days) + 75 mg/m2 AZA for 7 days per 28-day cycle induced CR and CRi in 29.9% and 50.5% patients with a 26-month median OS in naïve HR MDS [150]. The survival benefit of this combination is waiting for confirmation in the phase 3 VERONA study. Further optimization of the dose and schedule might still be needed because grade 3/4 neutropenia, thrombocytopenia, and febrile neutropenia were still observed in 48.6%, 44.9%, and 42.1% of patients, respectively.

#### Targeting stem cell protein TIM-3 for MDS treatment

TIM-3 is expressed on LSCs and MDS-SCs but not on normal HSCs. In AML, TIM-3 promotes an autocrine stimulatory loop via the TIM-3–galectin-9 interaction, which supports LSC self-renewal and disease progression [151]. Production of the TIM-3 ligand galectin-9 is increased in patients with MDS, which may contribute to disease progression by stimulating self-renewal in MDS-SCs [152]. HMA + SAB induces an OR in 56.9% of patients with HR- and very HR-MDS [153]. Median response duration for such treatment is 17.1 months and the 12-month progression-free survival (PFS) rate is 54.0% [153]. In January, 2024, the sabatolimab trial was terminated by the manufacturer due to failure to meet its primary endpoint.

#### Targeting CD47 for MDS treatment

Like many types of cancer cells, MDS-HSPCs express high levels of CD47 and hijack this signal to escape immune phagocytic destruction. MAG, an anti-CD47 monoclonal antibody, increases phagocytosis of tumor cells by blocking CD47 inhibitory signaling. In the ENHANCE randomized phase III trials, HMA + MAG induced CR in 33% of patients and ORR in 75% of them, with a median duration of OR and PFS of 9.8 and 11.6 months, respectively. Importantly, such treatment induced a 40% CR with a median OS of 16.3 months in *TP53*-mutant patients [154]. However, careful analysis of the data from the ENHANCE-2 and ENHANCE-3 trials reveals that HMA + MAG treatment increased the risk of anemia and death, leading the FDA to halt HMA + MAG treatment for MDS in February, 2024.

#### Reactivation of TP53 for treatment of TP53-mutant MDS

*TP53* mutations have been detected in 5–10% of MDS patients. Biallelic *TP53* mutations or VAF ≥ 40% *TP53* mutation predict significantly worse outcomes [155]. EPR is a first-in-class, small-molecule p53 activator which binds to mutant TP53 molecules to stabilize and adjust their structure into an active conformation [156]. Two phase 1b/2 studies showed that an AZA + EPR combination induced ORR in 62–73% patients, CR in 47–50% patients, and cytogenetic response (defined as a *TP53* VAF of < 5%) in 58% cases with a median duration of response of 10.4–12.1 months [104, 105]. AEs were similar to those reported for AZA or EPR monotherapies. Both studies suggested a better CR rate in the AZA + EPR combination than with monotherapy using AZA alone [104, 105, 155]. In March, 2022, the FDA approved EPR as an orphan drug and assigned it a fast-track designation for both MDS and AML. However, a phase 3 trial of the EPR + AZA combination did not show benefits superior to single-agent AZA treatment, most likely due to lack of biomarkers for proper patient selection [157].

#### Potential solutions

For all the above combination treatment regimens, both managing myelosuppressive symptoms and selecting proper patient subsets are critical for future clinical trials. The treatment effect of HMA is dependent on DNMT1 depletion. In fact, 0.1–0.2 mg/kg/d. (∼5 mg/m^2^/d.) of DEC is sufficient to induce DNMT1 depletion in target cells [94, 95]. But the current standard dose to treat HMAs is excessively high, impairing self-renewal of normal HSCs and inducing myelosuppression in patients. Most other agents used in combinations also cause myelosuppression, which exacerbates the adverse hematopoietic events induced by HMAs. Thus, future studies are needed to determine whether long-term, lower dose AZA might provide better responsiveness when combined with other agents. In addition, some agents such as EPR inhibit cell cycle progression, antagonizing the cell cycle-dependent activity of HMAs. In such cases, sequentially using AZA and EPR to avoid cell cycle arrest at the time of AZA administration or addition of G-CSF in the regimens to induce cell cycle progression of mutant cells might enhance the responsiveness of mutant cells to AZA treatment and alleviate the myelosuppressive-associated AEs.

For patient selection, a better understanding of the molecular mechanisms by which different medications work and elucidating the detailed pathogenesis of different subtypes of MDS are required. For example, a preclinical study of MDS suggested that patients with a less differentiated common myeloid progenitor (CMP) pattern are responsive to HMA + VEN treatment, whereas patients with a more differentiated granulocyte-monocyte progenitor (GMP) pattern are resistant due to the activation of TNFα-NFκB-MCL1 signaling [158]. *STAG2* or *RUNX1* mutations are enriched in patients with GMP pattern. In patients with the CMP pattern, after HMA + VEN induced remission, relapse and refractoriness occurred shortly in patients with MDS-SCs containing *TP53* mutations or obtained a GMP-transcriptional differentiation state or expansion of clones with *STAG2* or *RUNX1* mutations [158]. Thus, HMA + VEN treatment might only benefit patients with a CMP pattern. For the HMA + SAB and HMA + MAG combinations, more detailed analyses of TIM-3 and CD47 expression in both mutant cells and normal hematopoietic cells are needed in order to select the cases that have high levels of TIM-3 and CD47 expression in a large majority of mutant cells but not in healthy HSPCs. For the AZA + EPR combination, *TP53* mutational status might not be a reliable biomarker for patient selection. A recent in vitro screening study demonstrated that a low level of SLC7A11 expression is a better biomarker to predict EPR sensitivity but not *TP53* mutational status [159]. In addition to *TP53*, *SLC7A11* expression is also regulated by ATF4, MDM2, c-Myc, and ARID1A [160]. Future clinical trials need to determine whether SLC7A11 levels in *TP53*-mutant cases can be a reliable biomarker to predict AZA + EPR treatment responses and OS in MDS patients.

### Lack of reliable systems to evaluate novel treatments preclinically

Although in vivo animal xenografts provide better models for the preclinical evaluation of drug treatments, the generation of MDS patient-derived xenografts (PDXs) is still a challenge, despite improvements in animal modeling [161]. In in vitro cultures, MDS-HSPCs grow slowly in suspension medium due to increased PCD and abnormal differentiation [162]. As is true for normal HSCs, MDS-HSCs in such systems can only be sustained for several days, followed by rapid exhaustion. In methylcellulose medium, MDS-HSPCs frequently fail to show the presence of colony-forming units (CFUs) [163]. Most patient samples grow fewer CFUs, and many samples grow small clusters of granulocytes/monocytes, a feature shared with AML samples [163]. Thus, better in vitro culture systems, such as organoid cultures, are urgently needed to be able to reliably evaluate novel drugs preclinically [164].

### The molecular mechanisms by which EPOR signaling is impaired in MDS patients have not been elucidated

Normal erythropoiesis is tightly regulated by signals emanating from BM niches and EPO, which collaboratively promote the self-renewal and expansion of BFU-Es, survival and timely differentiation of CFU-Es, and multiple stages of erythroblasts. Impaired self-renewal of BFU-Es, increased PCD, and disrupted differentiation of erythroblasts all can cause ineffective erythropoiesis and anemia. A recent study suggested that the activation of muscarinic acetylcholine receptor CHRM4 signaling may impair the self-renewal of BFU-Es in some MDS patients [165]. The inhibition of CHRM4 promotes BFU-E expansion by inducing the CREB-mediated expression of critical erythroid regulators including GATA2, ZFP36L2, and KIT. This study suggests that the inhibition of CHRM4 signaling could potentially be a new approach for anti-anemia therapy in some MDS cases [165].

Normal sEPO levels in healthy controls are 4–21 IU/L [166]. In MDS, > 83% of patients have increased sEPO levels, ranging from 26–4530 IU/L [167]. In patients who respond to ESA treatment, partially impaired EPOR signaling in their erythroid precursors and early erythroblasts might be a major cause of anemia. In such patients, strong EPOR stimulation is required for its full activation. Uncovering the molecular mechanism by which EPOR signaling is impaired in such patients will help to further improve the treatment effects and prevent relapse and ESA resistance. In patients that do not respond to ESA treatment, determining how EPOR signaling is severely impaired will help to develop novel approaches to treatment. EPOR signaling can be disrupted by either an alteration of the key mediators of the pathway or by an interruption in inflammatory cytokine signaling. For example, IFNα impairs erythropoiesis by inducing the expression of cytokine-inducible SH2–containing protein (CISH), a negative regulator of EPO/EPOR signaling [168]. Other inflammatory cytokines, such as TNFα and IL1β, might also antagonize EPOR signaling [169]. Thus it is important to determine whether the ESA treatment response in MDS patients is associated with serum levels of these inflammatory cytokines.

### Effective treatments for patients with thrombocytopenia and neutropenia have not been developed

More than 10% of MDS patients develop severe thrombocytopenia (< 20 × 10^9^/L platelets) and experience potentially deadly bleeding episodes. For such patients, in addition to platelet transfusion [170], the TPO-RAs (thrombopoietin receptor agonists) romiplostim and eltrombopag have been tested for induction of platelet production. In LR patients, TPO-RA treatment improved platelet counts and reduced bleeding events in 36–46% of cases [171–175]. A recent long term phase II randomized controlled trial demonstrated that among responders to eltrombopag treatment, 25.5% of them eventually lost responsiveness while 63.6% of responders still maintained thrombocytopenic relapse-free survival at 60 months [174]. These studies suggested that TPO-RAs are relatively safe with promising efficacy in LR-MDS with severe thrombocytopenia. Durable responses were observed in ~ 25% of patients. However, there is a concern for increasing rates of AML transformation and marrow fibrosis in IR and HR patients [172, 176, 177]. Thus, TPO-RAs are only limited to RS and del(5q)-negative LR patients with severe chronic thrombocytopenia. Currently we are lacking reliable biomarkers to predict TPO-RA treatment responses. There is only one study which suggested that patients with *SRSF*2 mutations exhibited better responses than seen in patients with wild-type *SRSF2* [178]. For IR and HR patients, low-dose HMAs might improve platelet counts in some cases [45]; however, the addition of eltrombopag to the HMA regimen worsened platelet recovery and trended toward increased progression to AML [179]. Furthermore, IST with androgenic agents such as danazol might also improve platelet counts in some MDS patients [180, 181]. Danazol induces platelet improvement in 45% of LR-MDS cases [180]. Until now, no specific treatment has been established for MDS-related neutropenia as G-CSF is mostly reserved for patients with febrile neutropenia [182].

## Searching for new vulnerabilities for selective elimination of disease clones

### Targeting innate immune signaling pathways for MDS treatment

Many genetic abnormalities and epigenetic events in MDS enhance sensitivity of mutant HSPCs to TLR ligand stimulation. For example, haploinsufficiency of the intrinsic TRAF6 regulators *miR-146a* and *TIFAB* in del(5q) MDS and elevated expression of the IRAK4-L isoform in *URSF2-*mutant MDS led to over-activation of the TLR-MYD88-IRAK-TRAF6 pathway [183, 184]. In addition, several TLRs and their downstream effectors (*e.g.,* IL-1RAP,TIRAP, MYD88, IRAK1*,* and TRAF6) are up-regulated in CD34^+^ HSPCs and malignant myeloid cells of some MDS patients [184, 185]. This pathway activates TAK1-mediated IKK-NFκB, JNK, and p38 MAPK-mediated proliferation, survival, and inflammation. Thus, the inhibition of the TLR-MYD88-IRAK-TRAF6 pathway has been proposed for MDS treatment. For example, the anti-TLR2 antibody tomaralimab (OPN-305) induced an ORR in 50% of LR-MDS cases after HMA failure with a minimum reduction in TD in an early clinical trial [167]. The dual IRAK4 and FLT3 inhibitor emavusertib (CA-4948) induces marrow CR in 57% (4/7) of spliceosome-mutated HR-MDS when combined with AZA or VEN [186]. Currently, both tomaralimab and emavusertib are progressing to phase 2 trials.

### Targeting the splicing machinery for treatment of spliceosome-mutant MDS

Preclinical studies suggested that HSPCs with spliceosome mutations are hypersensitive to splice modulator treatment compared to healthy HSPCs, and inhibition of the splicing machinery will preferentially kill spliceosome-mutant HSPCs [187]. Currently, splice modulators such as H3B-8800 are in early clinical trials for spliceosome-mutant MDS [188].

### Targeting PANoptotic signaling for MDS treatment

Innate immune responses are always associated with inflammatory types of cell death, such as extrinsic pyroptosis, apoptosis, and necroptosis. In certain situations, all three types of PCD can occur simultaneously. It is referred to as PANoptosis, and it is caused by the dysregulation of the master regulators of the PANoptosome. Recently, we found that some of the master regulators of the PANoptosome are dysregulated in the HSPCs from some MDS patients. For example, two negative regulators of PANoptosomes, TAK1 and Caspase 8, are mis-spliced and downregulated in *SF3B1*- and *SRSF2*-mutant HSPCs, respectively. Consequently, we detected increased spontaneous PANoptosis in BM HSPCs from such gene-mutant MDS patients (Zhang et al*.*, Blood Advances, in press). We also found increased spontaneous PANoptosis in patients with many other types of mutations. Our study suggests that targeting the PANoptotic pathway might be a novel strategy to improve treatment for these patients.

### Targeting DNA damage response and repair pathways for MDS treatment

Dysregulation of ATM/ATR- and PARP-mediated DNA damage response signaling has been reported in MDS patients [189]. Spliceosome mutations induce R-loops into the RNA of HSPCs leading to hyper-sensitivity of the mutant HSPCs to ATR inhibition, which may induce synthetic lethality in spliceosome-mutant HSPCs [189]. Synthetic drug screens found that *SF3B1*^*mut*^ cells are sensitive to PARP inhibition [190]. Targeting ATR or PARP signaling might be a novel way to treat MDS with spliceosome mutations, specifically when combined with HMA or imetelstat treatment.

## Conclusion: step-by-step toward curing MDS

Treating MDS remains challenging due to its heterogeneity and the largely palliative nature of current treatments, aside from allo-HSCT. These treatments primarily address symptoms like anemia (Fig. 2a). As research continues, more therapeutic targets have been identified, leading to advances in targeted and combination therapies to eliminate mutant clones and prevent AML transformation. However, these new treatments show varying efficacy and toxicity (Fig. 2b). Emerging vulnerabilities, such as TLR signaling, PANoptosis, spliceosome mutations, and DNA damage response pathways offer potential avenues for novel therapies to be developed (Fig. 2c).

To develop personalized therapies for MDS patients, detailed characterization of the pathogenesis of different subtypes of MDS cases is essential. Recently, using IPSS-M to reanalyze data from previous clinical trials, researchers found that the risk groups re-stratified by IPSS-M better predicted prognostic accuracy among all MDS patients compared to IPSS-R, suggesting that IPSS-M should be used in the future for therapeutic decision-making [7, 19–21, 191]. Given the heterogeneous nature of MDS, future studies will need to further characterize the cellular and molecular pathogenesis of this disease by including not only the types and numbers of genetic mutations in each patient but also the clonal structure and differentiation hierarchy of the mutant clones. In addition, monitoring the dynamic changes of disease clones during treatment will provide insight into the underlying mechanisms by which the medications inhibit certain disease clones but may promote the selection of other clones for disease progression. Such efforts will instruct a joint effort by clinicians and researchers toward the development of targeted personalized therapies for patients based not only on the genetic abnormalities of their disease but also the clonal structures of their disease.

## Supplementary Information

Supplementary Material 1

## Data Availability

No datasets were generated or analysed during the current study.

## Abbreviations

Act-RIIB: Activin type IIB receptor
AE: Adverse effects
allo-HSCT: Allogenic hematopoietic stem cell transplant
AML: Acute myeloid leukemia
AML-MRC: AML with myelodysplastic type changes
ATG: Anti-thymocyte globulin
AZA: Azacitidine
BM: Bone marrow
CDA: Cytadine deaminase
CED: Cedazuridin
CFU: Colony-forming unit
CISH: Cytokine-inducible SH2-containing protein
CK1α: Casein kinase I α
CMP: Common myeloid progenitor
CR: Complete response
DEC: Decitabine
DNMT1: DNA methyltransferase 1
EPO: Erythropoietin
EPR: Eprenetapopt
ESA: Erythropoiesis-stimulating agent
GMP: Granulocyte-monocyte progenitor
Hb: Hemoglobin
HI: Hematopoiesis improvement
HMA: Hypomethylating agent
HR: High risk
HSC: Hematopoietic stem cell
HSPC: Hematopoietic stem and progenitor cell
HTB: High transfusion burden
IPSS-M: IPSS-molecular
IPSS-R: Revised IPSS
IR: Intermediate risk
IST: Immune-suppressive therapies
LEN: Lenalidomide
LMB: Low mutational burden
LR: Low risk
LSC: Leukemia stem cells
MAG: Magrolimab
MB: Mutational burden
MDS: Myelodysplastic syndromes
MDS-EB: MDS with excess blasts
ORR: Overall response rate
OS: Overall survival
PB: Peripheral blood
PCD: Programmed cell death
PDX: Patient-derived xenograft
PFS: Progression-free survival
PR: Partial remission
R/R: Relapsed and refractory
RA-RS: Refractory anemia with ring sideroblasts
RBC: Red blood cell
RS: Ring sideroblasts
s.c.: Subcutaneous
SAB: Sabatolimab
SC: Stem cell
sEPO: Serum erythropoietin
TB: Transfusion burden
TD: Transfusion-dependent
TI: Transfusion independence
TPO-RA: Thrombopoietin receptor agonist
VAF: Variant allele frequency
VEN: Venetoclax
