# Supplementary material for: Advances and challenges in the treatment of myelodysplastic syndromes
Source: Exp Hematol Oncol. 2025 Jun 18;14:87. doi: 10.1186/s40164-025-00678-9 (PMC12177969; doi:10.1186/s40164-025-00678-9)
Supplement: Supplementary file 1 — Supplementary Material 1 [file 40164_2025_678_MOESM1_ESM.docx]

**Supplementary Table 1. WHO-5^th^ edition and ICC classifications for MDS**

**
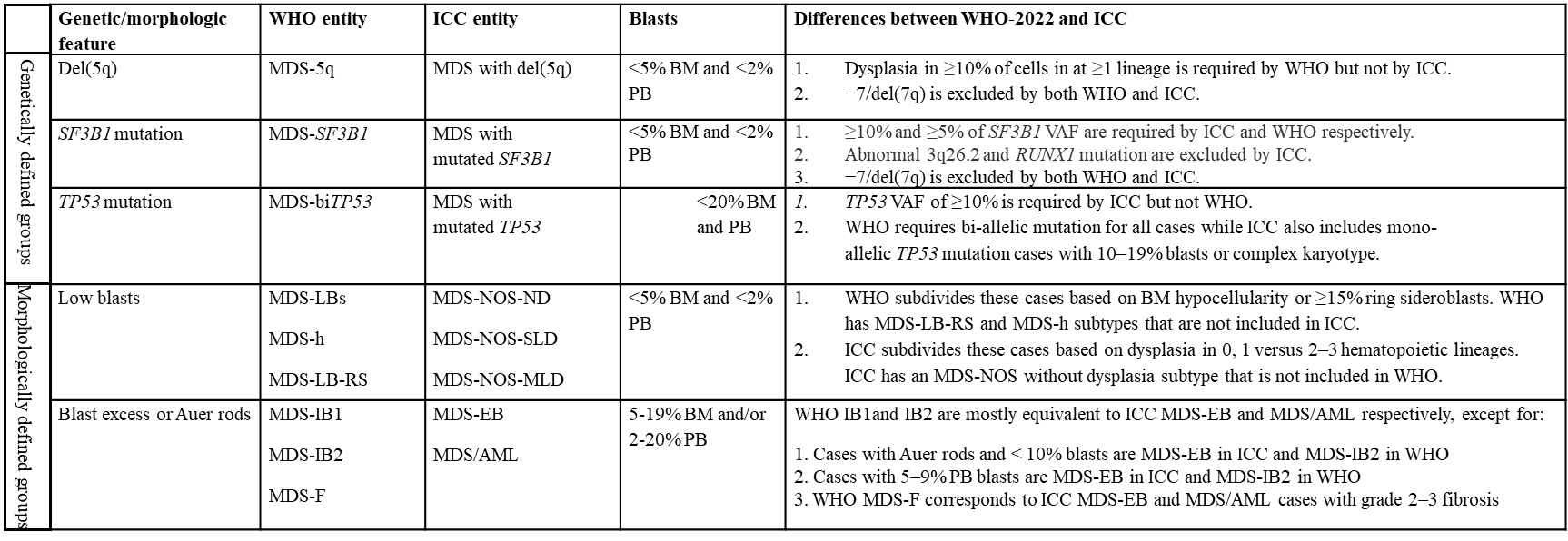
**

Abbreviations. MDS-5q: MDS with isolated 5q deletion; MDS-*SF3B1:* MDS with low blasts and *SF3B1* mutation; MDS-bi*TP53:* MDS with biallelic *TP53* inactivation; MDS-LBs: MDS with low blasts; MDS-h: MDS-hypoplastic; MDS-LB-RS: with low blasts and ring sideroblasts; MDS-IB1: MDS with increased blasts-1; MDS-IB2: MDS with increased blasts-2; MDS-F: MDS with increased blasts and fibrosis; MDS-NOS-ND: MDS, NOS without dysplasia; MDS-NOS-SLD: MDS, NOS with single lineage dysplasia; MDS-NOS-MLD: MDS, NOS with multilineage dysplasia; MDS-EB: MDS with excess blasts.

Both 5^th^ edition of the WHO classification (WHO-2022)^1^ and International Consensus Classification (ICC)^2^ classify MDS into three genetically-defined groups (SF3B1, TP53, or del(5q)) based on defining genetic abnormalities and two morphologically-defined groups (no blast excess and blast excess) based on the cutoff blasts% in BM (5%) and/or in PB (2%). WHO further subdivides no blast excess cases based on BM hypocellularity or ≥15% ring sideroblasts, while ICC subdivides these cases based on dysplasia in 0–3 hematopoietic lineages. Hypoplastic MDS (MDS-h) is defined by age-adjusted hypocellularity (cellularity < 20% for patients ≥70 years and < 30% for patients < 70 years). The threshold for defining dysplasia is recommended as 10% for all lineages. The WHO-2022 has also retained ring sideroblasts in the absence of *SF3B1* mutation as a morphologically-defined entity, although recent studies have shown similar prognosis to cases of MDS with low blasts that lack ring sideroblasts. Although the blast cutoff of 20% for the definition of AML remains, several specific AML-defining genetic lesions are considered to diagnose AML with ≥10% BM or PB blasts, including *PML*:*RARA,* other *RARA*rearrangements, *RUNX1:RUNX1T1, CBFB:MYH11, MLLT3::KMT2A,* other *KMT2A*rearrangements*, DEK:NUP214, GATA2; MECOM(EVI1),* other*MECOM*rearrangements, mutated*NPM1,* and in-frame *bZIP CEBPA* mutations.

1 Khoury, J. D. *et al.* The 5th edition of the World Health Organization Classification of Haematolymphoid Tumours: Myeloid and Histiocytic/Dendritic Neoplasms. *Leukemia* **36**, 1703-1719 (2022). <https://doi.org/10.1038/s41375-022-01613-1>

2 Arber, D. A. *et al.* International Consensus Classification of Myeloid Neoplasms and Acute Leukemias: integrating morphologic, clinical, and genomic data. *Blood* **140**, 1200-1228 (2022). <https://doi.org/10.1182/blood.2022015850>
